# Dental Legislation

**Published:** 1884-01

**Authors:** 


### EDITORIAL ETC.

DENTAL LEGISLATION.—The two bills now before the Legislature of the State of Maryland read as follows, the accompanying Circular showing the principal points wherein they differ:

MARYLAND STATE DENTAL ASSOCIATION,

DR. T. J. SMITHERS, of Easton, *President.*

DR. B. MERRILL HOPKINSON, of Baltimore, *Rec. Secretary.*

*A bill Entitled on Act to regulate the Practice of Dentistry, and to protect the people against Empiricism in relation thereto in the State of Marylary.*

SECTION 1. *Be it enacted by the General Assembly of Maryland.* That it shall not be lawful for any person or persons to engage in the practice of dentistry in the State of Maryland, unless said person or persons have graduated and received a diploma from the faculty of a Dental College or a Dental Department connected with a Medical University or Medical College, chartered under the authority of one of the United States of America, or from a foreign Government, or who at the time of the passage of this Act are engaged in active practice of dentistry, or shall have obtained a license from a Board of Examiners, duly authorized and empowered by this Act to issue such license.

SEC. 2. *And it be enacted,* That the said Board of Examiners thall consist of five dentai graduates, who are members in good sarnding of the Maryland State Dental Association, provided that said graduates have been practicing in the State of Maryland for a term not less than three years; the said Board of Examiners shall be appointed by the Maryland State Dental Association at their next annual meeting, or so soon as they may deem it necessary, who shall serve for a term of three years, when their successors shall be appointed, and every third year thereafter, unless otherwise provided by law; the said Board shall have power to fill all vacancies in said Board, for unexpired terms.

SEC. 3. *And be it enacted,* That it shall be the duty of said Board: *first*, to meet annually at the time of meeting of the Maryland State Dental Association, or oftener, at the call of any three members of said Board; thirty days notice must be given at the annual meeting; *secondly*, to grant a license to any applicant who shall furnish satisfactory evidence of having graduated and received a diploma from any reputable and incorporated Dental College, or University, or who at the time of the passage of this Act is engaged in the active practice of dentistry, without fee, charge, or examination ; *thirdly*, to grant license to all other applicants who undergo a satisfactory examination before said Board ; for which a fee of $5.00 shall be charged, the same fee to be applied by the Maryland State Dental Association for the expense of thc Examining Board : *fourthly*, to keep a book, in which shall be registered the names of all persons licensed to practice dentistry in the State of Maryland.

SEC. 4. *And be it enacted*, That the book so kept, shall be a book of record, and a transcript from it, certified to by the officer of said Board having it in keeping, with the seal of said Board attached thereto, shall be evidence in any Court in the State.

SEC. 5. *And be it enacted*, That three members of said Board shall constitute a quorum for the transaction of business, and should a quorum not be present on the day appointed for their meeting those present may adjourn from day to day, until a quorum is present.

SEC. 6. *And be it enacted*, That one member of said Board may grant a license to any applicant to practice dentistry, until the next regular meeting of the Board, when he shall report the fact, at which time, the temporary license shall expire ; but such temporary license, shall not be granted by a member of said Board, after the Board has rejected the applicant.

SEC. 7. *And be it enacted*, That any person or persons who shall in violation of this Act, practice dentistry in the State of Maryland for a fee or a reward, shall be deemed guilty of a misdemeanor, and upon conviction thereof, shall be punished by a fine of $100.00 or not more than $500.00, and be imprisoned in jail not less than one month or more than one year ; all fines received under this Act to be paid into the Common School Fund of the county in which such conviction takes place ; *provided*, that none of the provisions of this Act shall apply to regular licensed physicians or those holding diplomas, and surgeons in practice, or dentists who are in active practice at the time of the passage of this Act.

SEC. 8 *And be it enacted*, That every person practicing dentistry in this State, shall within ninety days after the passage of this Act, register his name, together with his post office ———, and the date of his diplomas or license in the office of the Clerk of the Circuit of the county or Court of Common Please of the city in which he practices, and shall on the payment to such Clerk a fee of fifty cents, be entitled to receive from him a certificate of such registration.

SEC. 9. *And be it enacted*, That all laws, and parts of laws, in conflict with this Act be, and the same are hereby repealed.

SEC. 10. *Be it enacted*, That this Act shall take effect from the date of its passage.

NOTE.—The following States have laws regulating the practice of dentistry; Alabama, Georgia, Illinois, Indiana, Iowa, Kentucky, Louisiana, Michigan, Missouri, Mississippi, New Hampshire, New Jersey, New York, North Carolina, Ohio, Pennsylvania, South Carolina, Vermont, West Virginia.

---

BALTIMORE, JANUARY 1, 1884.

DEAR SIR;

At the annual meeting of the "Maryland State Dental Association," held October 18, 1883 a Committee was appointed to draft a Bill to regulate the practice of dentistry in the State of Maryland, and at an adjourned meeting, held November 8, 1883, this Committee reported the enclosed Bill, which was unanimously adopted, and a Committee appointed to present the same to the State Legislature at the Session of 1884.

The "Maryland State Dental Association, " from which this Bill emanates, is the only legally chartered State Association in Maryland, and is composed of a large number of the most respectable dentists of the State.

Two months after the action of the State Association, a Society, formed by some dentists under the title of the "Dental Legislative Society, " perfected a Bill, which they propose to present to the Legislature also during the present session, and merely for the purpose of opposing the action previously taken by the State Association.

While the Bill adopted, and to be presented to the Legislature by the " Maryland State Dental Association, " meets all the requirements, and is not in the least oppressive, the proposed Bill of the " Dental Legislative Society," should it become a law, will not only be oppressive in its action, but will render null and void the charters granted by the Legislatnre of Maryland to Dental Institutions of learning, insomuch that it gives a few dentists, who may be altogether unfit to judge, the power to pass upon diplomas granted by such institutions, and which they are authorized to grant by a State Law. Thıs proposed Bill of the "Dental Legislative Society" is also more oppressive, and imposes higher registration and other fees than the Bill proposed by the State Association'

The Bill of the State Association makes no provision whatever for paying fees to the members of an examining board, whereas the Bill of the "Dental Legislative Society" proposed to pay the members of such a board the sum of $5.00 each per meeting, without limiting the number of such meetings.

Should the Legislature of Maryland pass such a Bill as that proposed by the "Dental Legislative Society," the anomally of such action would be apparent, when the acts relating to the charters of the Dental Institutions of learning are considered, which acts not only empower the granting of diplomas by such institutions undcr certain conditions, but also authorize those holding them to practice dentistry in the State of Maryland free of all such restrictions as the Bill in question advocates.

In order that the points of difference existing between the two prospoed Bills may be fully understood, the Committee of the "Maryland State Dental Association" respectfully address to you this circular, feeling confident that the Bill proposed by this State Association is the only one that will accomplish the desired object.

RESPECTFULLY, &c.,

DR. F. J. S. GORGAS,
DR. D. F. PENNINGTON,
DR. M. A. HOPKINSON,
DR. A. C. MCCURDY,

*Committee of "Maryland State Dental Association."*

---

The following is the Bill presented by the "Dental Legislative Society:"

*An Act to insure the better education of practitioners of Dental Surgery, and to regulate the practice of Dentistry in the State of Maryland.*

SECTION 1. *Be it enacted by the people of the State of Maryland represented in the general assembly*, That it shall be unlawful for any person who is not, at the time of the passage of this act, engaged in the practice of dentistry in this State, to commence such practice, unless he obtaine a certificate as hereinafter provided.

SEC. 2 .A Board of Examiners, to consist of five practicing dentists, is hereby created, whose duty it shall be to carry out the purposes and enforce the provisions of this act. The members of such board shall be appointed by the governor, who shall select them from ten candidates, whose names shall be furnished him by the Dental Legislative Society of Maryland, provided that none of said ten candidates shall be pecuniarily connected with any dental college, or dental department of any college or university. Three members at least of this board shall be members of the Dental Legislative Society of Maryland. The term for which the members of said board shall hold their offices, shall be for five years, except that the members of the board first to be appointed under this act shall hold their offices for the term of one, two, three, four and five years respectively, and until their successors shall be duly appointed. In case of a vacancy occuring in said board, such vacancy shall be filled by the governor, from the names presented to him by the Dental Legislative Society of Maryand. It shall be the duty of the Dental Legislative Society of Maryland to present to the governor twice the number of names of those to be appointed.

SEC. 3 Said board shall choose one of its members president, and one secretary thereof. It shall fix the time and place of its regular meeting or meetings, and may meet as much oftener and at such times and places as it may deem necessary. A majority of said board shall, at all times, constitute a quorum, and the proceedings thereof shall, at all reasonable times be open to the public inspection. The board shall also make an annual report of its proceedings to the Dental Legislative Society of Maryland.

SEC. 4. Within six months from the time that this act takes effect, it shall be the duty of every person, who is at that time engaged in the practice of dentistry in this state, to cause his or her name and residence or place of business to be registered with said Board of Examiners, who shall keep a book for that purpose. The statement of every such person shall be verified under oath, before a notary public or justice of the peace, in such manner as may be presented by the Board of Examiners. Every person who shall so register with said board, as a practitioner of dentistry may continue to practice the same as such, and shall receive a certificate of such registration upon his paying to said board one dollar for such certificate. It shall be the duty of the Board of Examiners to forward to the county clerk of each county in the State or the clerk of the Court of Common Please of Baltimore, a certified list of the names of all persons residing in his country, or in said city, who have registesed in accordance with the provisions of this act ; and it shall be the duty of all said clerks to register such names in a book to be kept for that purpose.

SEC. 5. Any and all persons who shall so desire may appear before said board at any of its regular meetings, and be examined with reference to their knowledge and skill in dental surgery : and if the examination of any such person or persons shall prove satisfactory to said board, the Board of Examiners shall issue to such persons as they shall find to possess the requisite qualifications, a certificate to that effect, in accordance with the provisions of this act. Said board shall also endorse as satisfactory diplomas from any reputable dental college, when satisfied with the character of such institution, upon the holder of such diploma furnishing evidence satisfactory to the board of his or her right to the same, and shall issue a certificate of such endorsement to said holder, and shall register the names of such persons receiving certificates under this system, and forward them to the clerks of the counties, and Court of Common Please of Baltimore, as provided in section four of this act. All certificates issued by said board shall be signed by its officers, and such certificates shall be *prima facia* evidence of the right of the holder tc practice dentistry in the State of Maryland.

SEC. 6. Any person who shall violate any of the provisions of this act shall be deemed guilty of a misdemeanor, and upon conviction, may be fined not less than one hundred dollars or more than three hundred dollars, or to be confined six months in thc county jail. All fines received under this act shall be paid into the common school fund of the county in which such conviction takes place.

SEC. 7. In order to provide the means of carrying out and maintaining the provisions of this act, the said Board of Examiners may charge each person applying to, or appearing before them for examination for a certificate ot qualification, a fee of five dollars, and for every endorsement, if satisfactory, upon any diploma, a fee of two dollars, and out of the funds coming into the possession of the board from fees received by virtue of this act, each member of said board may receive as a compensation the sum of five dollars for each day actually engaged in the duties of his office, and all legitimate expenses incurred in attending the meetings of said board

SEC. 8. That one member of said board may grant any certificate provided for in this act, to any applicant, upon presentation by such applicant of the evidence requisite for the obtaining said certificate, which certificate shall remain in force until the next regular meeting of the said board after the granting of said certificate, and no longer; but no snch certificate shall be issued by such members after such applicant has been rejected by said board,

SEC. 9. Nothing in this act shall be so construed as to interfere with the rights and privileges of the physician and surgeon in the discharge of his or her professional duties.

SEC. 10. This act shall take effect from the date of its passage.

SEC. 11. That the Dental Degislative Society of Maryland be and is hereby incorporated.

The following named gentlemen were appointed a committee to submit this bill to your honorable body, and ask your favorable consideratlon. Respectfully submitted.

E. P. KEECH,
EDWARD. NELSON.
T. S. WATERS,
J. B. SUTHERLAND,
J. A. WEBB,
B. MYER,
*Committee.*

